# Rate of viral load change and adherence of HIV adult patients treated with Efavirenz or Nevirapine antiretroviral regimens at 24 and 48 weeks in Yaoundé, Cameroon: a longitudinal cohort study

**DOI:** 10.1186/s12879-019-3824-7

**Published:** 2019-02-26

**Authors:** Bih Hycenta Chendi, Marie Claire Okomo Assoumou, Graeme Brendon Jacobs, Elsie Laban Yekwa, Emilia Lyonga, Martha Mesembe, Agnes Eyoh, George Mondinde Ikomey

**Affiliations:** 1 Center for the Study and Control of Communicable Diseases (CSCCD), Faculty of Medicine and Biomedical Sciences, University of Yaoundé 1, Yaoundé, Cameroon; 2 Division of Medical Virology, Department of Pathology, Faculty of Medicine and Health Sciences, Stellenbosch University, Tygerberg, South Africa

**Keywords:** HIV-load, Adherence, Efavirenz, Nevirapine, HIV infected adult

## Abstract

**Background:**

HIV-load decrease and suppression over time is associated with consistent adherence to antiretroviral therapy (ART). Our study aimed to evaluate the difference in viral load and adherence of patients treated with a combination of either Tenofovir (TDF), Lamivudine (3TC) and Efavirenz (EFV) or TDF / Zidovudine (AZT), 3TC and Nevirapine (NVP) regimens at 24 and 48 weeks.

**Methods:**

A longitudinal study was conducted from May 2016 to June 2017 among 256 HIV infected adult patients who were enrolled at two approved treatment hospitals in Yaoundé, before the start of first-line ART. Whole blood samples were collected using standard operating procedures. HIV-loads were determined by a quantitative RealTime PCR assay. Adherence was evaluated by pharmacy refill data records. Statistical analyses were performed using the PRISM 5.0 software.

**Results:**

Off the 256 HIV infected patients enrolled, 180 (70%) patients completed the study and 76 (30%) patients were lost to follow-up. The success rate in achieving viral load < 40 copies/ml was 1.8 times higher with the EFV regimen at 24 weeks and was 1.2 times higher in the NVP regimen at 48 weeks. At 48 weeks the treatment failure rate was 12.0 and 40.0% in patients on EFV and the NVP regimen, respectively. The rate of adherence varied in both ART based regimens with 84.0 to 74.0% for EFV and 65.5 to 62.5% for NVP, at 24 and 48 weeks respectively.

**Conclusion:**

In our study and setting, the rate of viral load decrease was higher in the NVP based regimen than with the EFV regimen. The adherence rate to ART was higher in the EFV regimen, compared to the NVP regimen. This adds to evidence that the EFV regimen is the preferred ART combination for non-nucleoside reverse transcriptase inhibitors (NNRTIs).

## Background

The introduction of antiretroviral therapy (ART) world wide as treatment for HIV infections has greatly reduced mortality and morbidity among patients living with HIV [1, 2]. The first-line therapeutic scheme used in Cameroon involves the combination of two nucleoside reverse transcriptase inhibitors (NRTIs): Zidovudine (AZT) or Tenofovir (TDF) and Lamivudine (3TC); and a non-nucleoside reverse transcriptase inhibitor (NNRTI): either Efavirenz (EFV) or Nevirapine (NVP) [3]. Achieving favorable HIV treatment outcomes is a major challenge, particularly due to non-adherence and the development of strains harboring resistance associated mutations (RAMs) [4].

The standard approach for monitoring treatment outcomes in patients on ART depends on the measurement of HIV-load over time. According to the WHO guidelines, virological failure is observed when patients sustain a viral load > 1000 copies/ml after 6 to 12 months of ART. The persistent high viral load in most cases is due to non-adherence [5].

Studies from low- and middle-income countries have shown measuring adherence is more likely to predict virological failure than clinical and immunological criteria. In Cameroon, poor adherence, treatment interruption due to dosage and the loss to follow-up are factors reported most likely to influence virological failure responses. In spite of adherence being found to be an important factor in influencing treatment outcome, there are no standard guidelines for its measurement [6–10].

Cameroon follows and strictly applies the WHO ART guidelines with the EFV regimen (TDF, 3TC and EFV) taken as a single dose daily and the NVP regimen (AZT/TDF, 3TC and NVP) taken twice daily [3]. Though the EFV regimen was considered preferential over NVP in some studies; there are no clear-cut criteria or guidelines used in choosing either of the two regimens for patients at initiation of ART. This has raised many concerns over the correct ART combination to be provided at which time point. In addition, Cameroon is a resource limited country where historical ART access and economic and cultural limitations may still influence the optimal performance of ART. It is thus imperative to provide evidence with local data that will guide the clinicians to focus on the most effective treatment combinations. We, therefore, conducted a longitudinal study over 48 weeks on HIV-infected patients before receiving the two NNRTI-based regimens in Yaoundé, Cameroon.

## Methods

### Setting

A longitudinal cohort study was conducted from May 2016 to June 2017 to evaluate ART outcome at two approved treatment and care centers for HIV in Yaoundé (Yaoundé Central Hospital and “Centre Hospitaliered’Essos”). We determined the difference in viral load and assessed adherence in HIV adult patients.

### Study participants and data collection

The study population consisted of 256 consenting HIV infected adult patients who were all treatment naïve at the beginning of the study. Patients were initiated on either the combination of TDF/3TC/EFV (300 mg/300 mg/600 mg) taken as a single dose daily or AZT/3TC/NVP (300 mg/150 mg/200 mg) taken twice daily. Questionnaires were administered to collect socio-demographic data and treatment information. Whole blood (5 ml) was collected in Ethylenediaminetetraacetic acid (EDTA) collection tubes from each patient for viral load testing at baseline (prior to the start of ART), 24 and 48 weeks, using standard procedures. Laboratory analyses were done at the Center for the Study and Control of Communicable Diseases (CSCCD) of the University of Yaoundé I.

### HIV-load testing

RNA viral load testing were performed before the start of ART and at 24 and 48 weeks of treatment using an automated RealTime PCR assay:CobasAmpliprep/CobasTaqman 96 platform (Roche Diagnostics, Mannheim, Germany). Plasma samples were analyzed based on the manufacturer’s instruction. The detection limit of the assay was < 40 copies per ml.

### Measurement of adherence

There are no standard guidelines for the measurement of adherence to ART. Drug screening for adherence was evaluated based on pharmacy refill data records reporting the number of patients who came for their drugs on their respectful appointment dates. This enabled us to evaluate the number of patients who continued one of either EFV or NVP containing first-line NNRTI regimens during the study period as being adherent to ART. We classified patients’ rate of adherence as being good, fair, poor or non-adherent based on a rating scale we developed with a define limit of 40% and interval of 20%. The number of patients who continued treatment was converted to percentage in order obtain the rate of adherence. A rate ≤ 40% was considered non-adherent to ART, 40–60% as poor adherence, 60–80% as fair adherence and ≥ 80% as good adherence. At baseline, all patients were considered adherent at 100% to their respective ART regimens.

### Statistical analyses

Data were recorded in a Microsoft Excel sheet and statistical analyses were performed using the Graphpad PRISM 5.0 software package (GraphPad Software, Inc., La Jolla, California, USA). Descriptive statistics were used to analyze the patient’s demographic information. Repeated measures of the ANOVA test was computed to outline the mean difference in viral load. The Mann-Whitney non-parametric tests were used to calculate the mean difference in the level of detectable viral load. Relative risk ratio and percentage relative effect were used to predict the likelihood of achieving virological suppression. Different level approach namely: good, fair, poor and non-adherence was used to evaluate adherence according to pharmacy records and the proportion of patients who remained on treatment using cross contingence tables. The level of significance of all analyses was set at *p* < 0.05.

## Results

### Demographic and clinical characteristics

Of the 256 infected patients enrolled in the study, 180 (70%) patients completed the study with 76 (30%) patients lost to follow-up. Of the 180 patients, 72 (40%) were male and 108 (56%) were female, with 100 patients on the EFV regimen and 80 patients on the NVP regimen. The age of the participants ranged from 24 to 57 years, with the most representative age group ranging from 38 to 43 years old. The characteristics of the study population are shown in Table 1.

**Table 1:** Participants characteristics at 48 weeks on ART

Variable	Category	Frequency (%)
Outcome of participants	Completed	180 (70.3%)
	Lost to follow up	76 (29.6%)
Gender	Female	108 (60%)
	Male	72 (40%)
Age (years)	Median	39
	≤ 25 years	8 (4%)
	26 – 31 years	20 (11%)
	32–37 years	52 (29%)
	38–43 years	48 (27%)
	44–49 years	28 (16%)
	≥ 50 years	24 (13%)
ART regimen	EFV	100 (55.5%)
	NVP	80 (44.4%)

Abbreviations: *ART* Antiretroviral therapy, *EFV* Efavirenz, *NVP* Nevirapine

### Outcomes of HIV-load over 48 weeks of ART

**Fig. 1:**
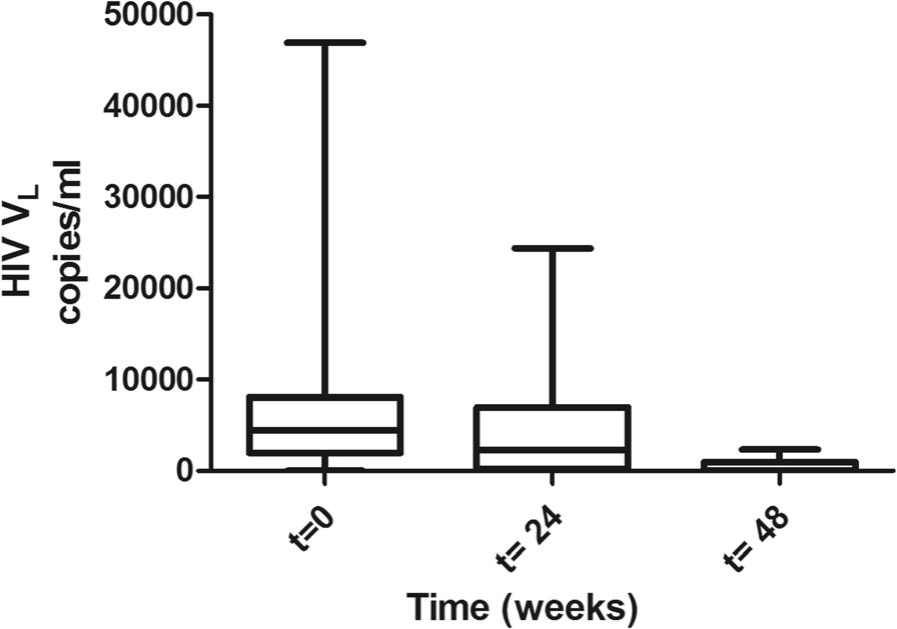
HIV-load over time. The Box plot shows the distribution of viral load in copies/ml at baseline, t = 0 before treatment, at 24 weeks (t = 24) and at 48 weeks (t = 48) on ART

**Fig. 2:**
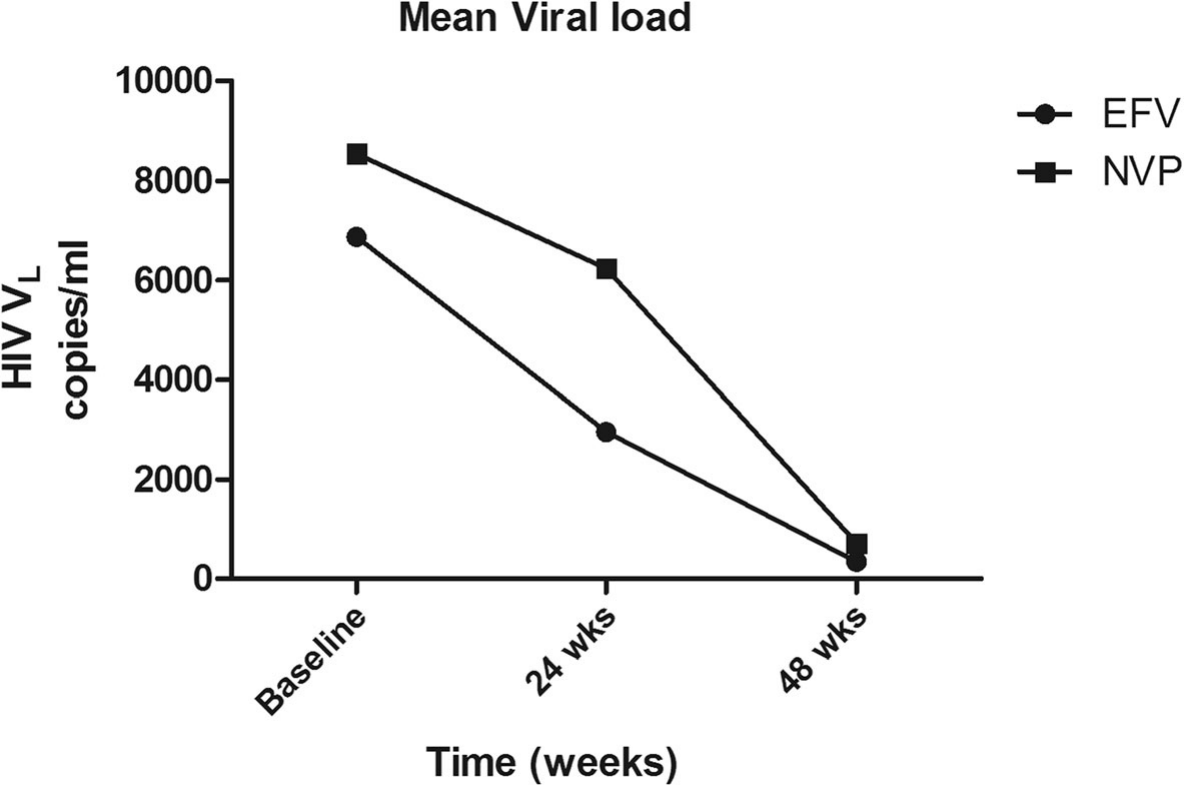
Difference in viral load. The variation of viral load in both efavirenz (EFV) and nevirapine (NVP) regimens were plotted at baseline, 24 and 48 weeks over time. Each point on the graph represents the mean viral load of patients at a given point in both regimens

There was a statistically significant decrease in HIV-load, from baseline to 48 weeks, among all regimens, with *p* < 0.0001. The median baseline HIV-loads were 4440, 2270 and 40 copies/ml at time points 0, 24 and 48 weeks, with semi interquartile ranges of 1976–8100, 204.8–7029 and 40–995, respective copies (Fig. 1). In both treatment regimens, there were a decrease in the mean value of the viral load. The HIV-load for patients on EFV at time points 0, 24 and 48 were 6871.0, 2954.8 and 335.2 copies/ml, respectively. For the NVP regimen, mean viral load at time points 0, 24 and 48 were 8546.0, 6237.3 and 715.1 copies/ml, respectively. The mean difference in viral load at each point time was significant in patients on either regimen with *p* = 0.0011 and *p* = 0.0055, respectively (Fig. 2).

### Virological suppression

The proportion of patients on either regimen who achieved a viral load result < 40 copies/ml at 24 weeks and 48 weeks were evaluated using the relative risk ratio and percentage relative effect. The analyses showed that at 24 weeks on ART, the possibility of achieving viral load < 40 copies/ml among patients taking the EFV regimen was 1.7 times higher than those on the NVP regimen (0.8). Viral load < 40 copies/ml was observed in 26.0% (*n* = 28/108) of patients on the EFV regimen as compared to 14.2% (*n* = 12/84) patients on the NVP regimen. Patients taking EFV had a 75.0% increased rate in obtaining a viral load < 40 copies/ml. Patients on the NVP regimen at 48 weeks were 1.2 times more likely to achieve a viral load of < 40 copies/ml with an increased possibility of 23.0%, compared to patients on the EFV regimen (0.5). The proportion of patients with viral load < 40 copies/ml at 48 weeks was observed in 60.0% (*n* = 48/80) of patients on the NVP regimen and in 52.0% (*n* = 52/100) of patients on the EFV regimen (Table 2).

**Table 2:** viral suppression of viral load < 40 copies/ml in both regimens. The probability of achieving viral load < 40 copies/ml was evaluated at 24 and 48 weeks in efavirenz and nevirapine regimen based on the relative risk ratio

	EFV		NVP	
	At 24 weeks	At 48 weeks	At 24 weeks	At 48 weeks
Patients (*n*)	28 (26.0%)	52 (52.0%)	12 (14.2%)	48 (60%)
Cumulative incidence	0.3	0.6	0.2	0.8
Relative Risk	1.7	0.8	0.5	1.2
% relative effect	75.0% increase	18.7% decrease	43.0% decrease	23.0% increase

Abbreviations: *EFV* Efavirenz, *NVP* Nevirapine, *n* number of patients

**Fig. 3:**
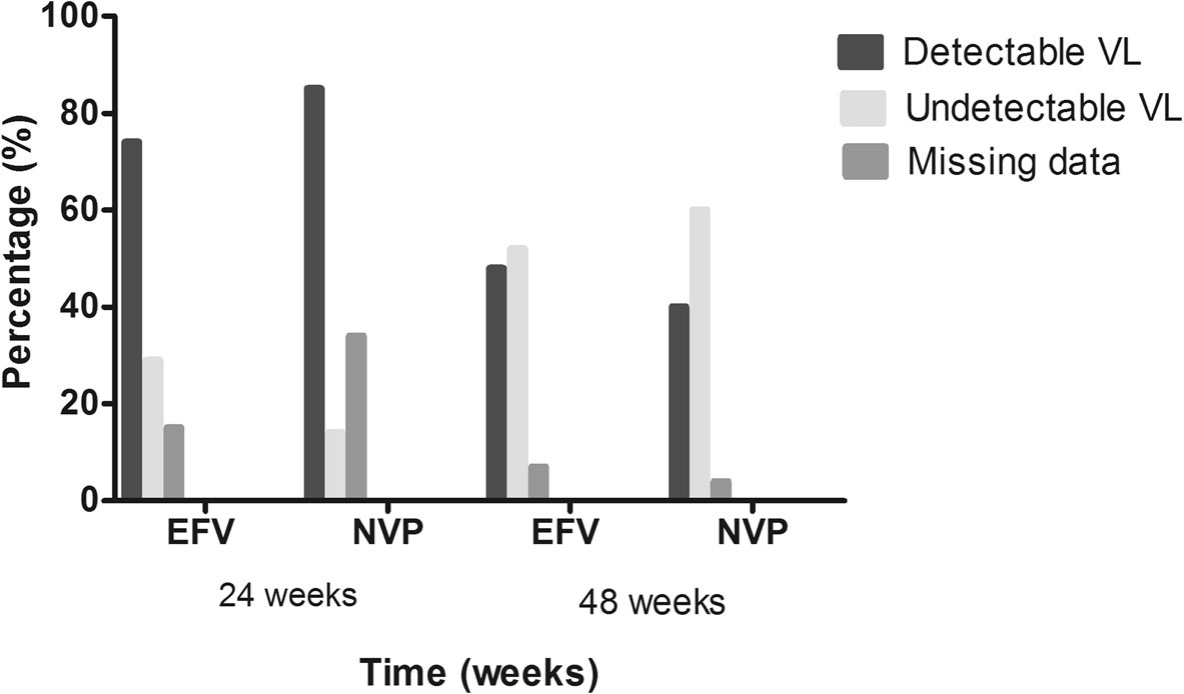
HIV-load suppression at 24 and 48 weeks. The level of viral load < 40 copies/ml and > 40 copies are represented as undetectable and detectable viral load levels in the chart. Each bar represents the percentage of undetectable and detectable viral load levels in both regimens at 24 and 48 weeks of treatment

There was a significant decrease in the mean level of detectable viral load (viral load > 40 copies/ml) from 24 weeks to 48 weeks, with *p* = 0.001 using the Mann-Whitney non-parametric test. In both regimens, EFV and NVP, the decrease in the viral load level was equally significant with *p* = 0.01 and *p* = 0.03, respectively. In addition, an increase in the level of undetectable viral load (viral load < 40 copies/ml) was attained from 24 weeks to 48 weeks in both regimens, with a greater increase level observed in the NVP group (from 14.2 to 60.0%) than with the EFV group (29.9 to 52.0%) (Fig. 3).

### Treatment failure

Sequential viral load monitoring is the recommended approach in defining treatment failure, despite its costly nature. We compared patients’ viral load at 24 and 48 weeks in each treatment regimen to evaluate treatment failure. Treatment failure was calculated based on two successive measurements of viral load with results > 1000 copies/ml, according to the WHO criteria. Data collected at 24 weeks were used as the baseline and that at 48 weeks was considered as the first time point to evaluate the virological failure rate. In patients on the NVP regimen, the rate of virological failure was higher, with 40.0%, than that of patients on the EFV regimen, with a rate of 12.0%. Twelve patients on the EFV regimen experienced treatment failure compared to thirty-two patients on the NVP regimen. Thus, patients on the EFV regimen were less likely to encounter treatment failure compared to those on the NVP regimen (Table 3).

**Table 3:** Treatment failure compare between regimen groups. Rate of sustainable viral load level > 1000 copies/ml at 24 weeks and at 48 weeks was calculated along side the median viral load*.* Virological failure rate decrease in both treatment regimens and was high in the NVP regimen compare to EFV

ART regimen	Rate of sustainable viral load level > 1000 copies/ml							
	Viral outcome at baseline		Viral outcome at 24 weeks			Viral outcome at 48 weeks		
	*n*	Median V_L_ (copies/ml)	*n*	Median V_L_ (copies/ml)	VFR(%)	*n*	Median V_L_ (copies/ml)	VFR(%)
EFV	80	6510	52	4440	48.1%	12	1345	12%
NVP	84	6930	64	6290	76.1%	32	1656	40%
Total	164	6930	116	6245	60.4%	44	1532	24%

Abbreviations: *EFV* Efavirenz, *NVP* Nevirapine, *VFR* Virological Failure Rate, *V*_*L*_ Viral load, *n* number of patients

### Adherence to ART

**Fig. 4:**
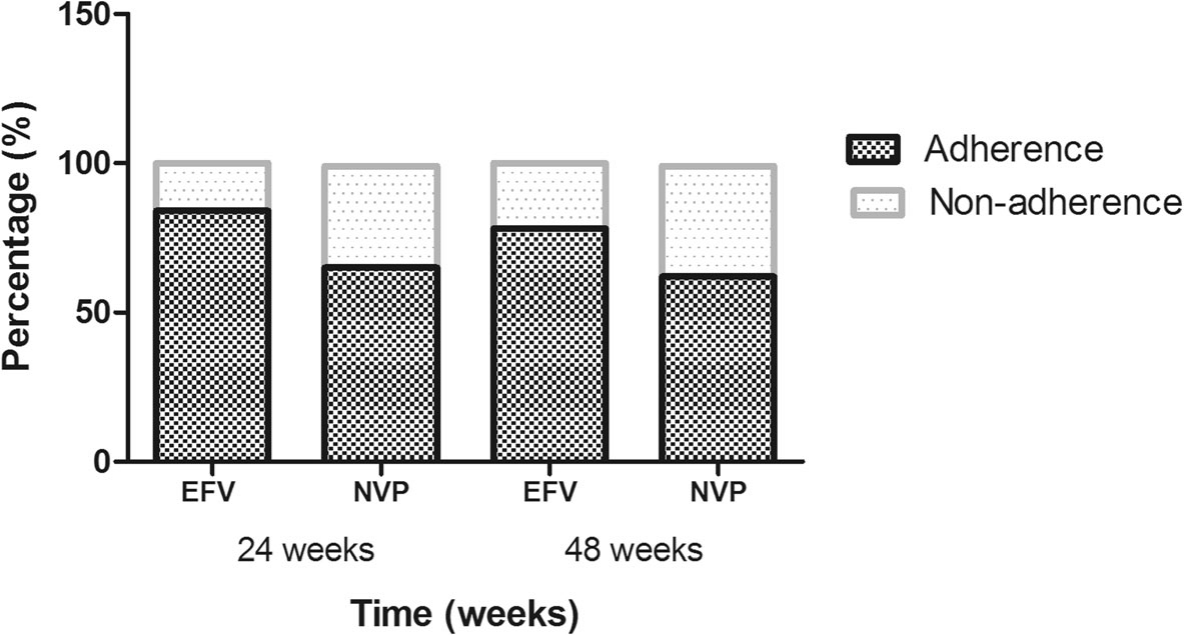
Adherence and non-adherence to ART regimens at 24 and 48 weeks. The percentage of patients considered adherent and non-adherent at 24 and 48 weeks, are represented in the chart. All patients on the EFV and NVP regimen were considered adherent at 100% at baseline

We evaluated the rate of adherence to ART regimens from pharmacy refill data, taking into consideration the number of patients still on treatment in both regimens. From 24 weeks to 48 weeks there was a decrease in the adherence rate and increase in non-adherent rate in both regimens. The proportion decreased from 84.0% to 78.0,% in patients taking the once-daily tablet EFV regimen and from 65.5 to 62.5% in patients taking the twice-daily tablet NVP regimen. The adherence rate was higher in the EFV group compared to the NVP group at 24 and 48 weeks, despite the decreased difference observed. The proportion of patients considered non-adherent increased from 16.0 to 22.0% in the EFV regimen and from 34.5 to 37.6% in the NVP regimen (Fig. 4).

Age group, gender and the difference in viral load was evaluated in relation to the rate of adherence. The age group 38–43 years was more representative among the group of patients with adherence to the EFV regimen. The most representative age group in the NVP regimen was 32–37 years. Females were more adherent with either regimen. More so, patients experiencing viral load changes were those adherent to their respective treatment regimens. (Table 4).

**Table 4:** Rate of adherence among age group, gender and viral load

	Adherence at 24 weeks			Adherence at 48 weeks		
	*n*	EFV	NVP	*n*	EFV	NVP
Age group (years)						
≤ 25	8	7.4%	/	8	8%	/
26–31	20	18.5%	/	12	12%	/
32–37	52	22.2%	33.3%	52	24%	35%
38–43	48	29.6%	19%	48	32%	20%
44–49	28	11.1%	19%	24	12%	15%
≥ 50	24	11.1%	14.2%	24	12%	15%
Gender						
Male	80	50%	47.6%	76	36%	45%
Female	112	59.2%	52.3%	104	60%	55%
VFR	116	48.1%	76.1%	44	12%	40%

Abbreviation: *EFV* Efavirenz, *NVP* Nevirapine, *VFR* virological failure rate, *n* number of patients

## Discussion

The evaluation and monitoring of the effectiveness of ART based on HIV-load is of great importance [11]. ART suppresses HIV-load, restores and improves immune function, thereby enhancing the quality of life [1, 12, 13]. The maintenance of the efficiency of ART requires some degree of high adherence, otherwise, it can inevitably lead to virological failure [14].

In this study, we observed a 70.0% retention rate from the 256 HIV infected adult patients recruited. This could be related to the limiting factors of a longitudinal study, such as lost to follow-up, change of contact, change of environment or even death. The 70.0% retention rate obtained was higher than that found by Dalal et al., (2015) and Alula et al., (2017) and raises concerns over poor maintenance of cohorts in longitudinal studies in Africa [15, 16]. Nevertheless, among the 30.0% lost to follow-up individuals, the majority of the patients were on the EFV regimen, more were men and the most representative age group range was 32–37 and 38–43 years.

We observed an overall significant decrease (*p* < 0.0001) in the mean value of HIV-load over time in both the EFV and NVP regimens (*p* = 0.0011 and *p* = 0.0055). This decrease in viral load was a result of the effectiveness of ART in preventing the replication of HIV. Our results are in accordance with the findings of Edward et al., (2015) who in their study in HIV-infected adults reported the effectiveness of ART in bringing about a dramatic decrease in viral load [17]. Similar results on the effectiveness of EFV and NVP were also obtained in a systematic review study aimed at determining the most effective NNRTIs when given in combination as part of ART [18].

We observed a significant increase (*p* = 0.0010) in the level of viral load < 40 copies/ml throughout the study, implying a progressive reduction in viral load at each time point. Patients on the EFV regimen at 24 weeks of treatment were better at achieving viral load < 40 copies/ml. At 48 weeks, patients on NVP had a better outcome in achieving the same viral load level. These results could be attributed to a difference in baseline HIV-load level in each regimen before ART initiation and treatment interruption during ART influencing the viral load. Hence, no constant decrease in HIV-load level. This finding is in accordance with the findings of Wu et al., (2015), in a five year longitudinal study evaluating the effectiveness of first-line ART in HIV/AIDS patients and with the findings of *Meresse* et al.*, (2013),* reporting the impact of treatment interruption on virological response [10, 11].

Patients taking the EFV combination were less likely to experience virological failure (48.0 and 12.0%), compared to the NVP regimen group (76.0 and 40.0%) at 24 and 48 weeks, respectively. The overall failure rate (24.0%) at 48 weeks of ART could be due to non-adherence in both treatment regimens. Similar results obtained in a cohort study in Uganda, South Africa and in a meta-analysis study reporting patients on EFV based regimens were less likely to experience virological failure than patients based on NVP regimens [19-21]. Our results are, on the contrary, slightly higher than that found by Zoofaly et al., in 2015 in a prospective cohort study in Cameroon reporting a 16.0% virological failure rate at 12 months. The study suggests the association of incomplete adherence with incomplete suppression of viral load [22].

In Cameroon till date, there are no clear-cut guidelines for the use of administrating one of either EFV or NVP containing first-line NNRTI regimens, despite the proven efficacy of the EFV regimen compared to the NVP-based regimen. While several studies in resource-rich settings have provided evidence of increased risk of virological failure associated with the NVP based regimen, only a few studies have been done to verify this hypothesis in resource poor settings [20, 21].

In this study, we observed good and fair adherence to ART at 24 and 48 weeks. Good adherence (81.0%) was observed in patients on the EFV regimen and probably resulted in the reduction in viral load. Fair adherence (64.0%) was detected among patients on the NVP regimen and might be associated with the high treatment failure. The high adherence rate in patients on the EFV regimen compared to the NVP regimen could be attributed to the dosing frequency difference in the regimens. Patients on the EFV regimen are administered a single dose, easy to administer, compared to patients on the NVP regimen taking a twice-daily dose. The good adherence rate to EFV regimen is in accordance with the findings of a cross-sectional survey that reported adherence variations of between 71.0 and 93.0% [7]. Similar observations of a decrease in adherence rates were also obtained in other studies reporting higher adherence with the once-daily regimen, compared to the twice-daily regimen [6, 9, 23]. Our results are, however, higher than those found in a study among Kenyan patients on long-term ART that reported good adherence at 55.8%, fair adherence at 22.2% and poor adherence at 22.0% [24]. Increase in non-adherence was equally observed and closer to previous findings performed in Cameroon. The outcome showed poor adherence as a result of dosing frequency inconveniences, patients neglecting to take ART, being away from home and concerns over privacy [4, 8, 25]. Nevertheless, compared to our evaluation system, most of the previous studies evaluating adherence were either based on self report questionnaires, drug concentrations in the blood, macrocytosis measurements, visual analogue scales, pill-counts and medication event monitoring systems (MEMS) [6, 7, 9, 23, 24].

In this study, the proportion of patients with reduced viral loads were found to be more adherent to ART and consisted of patients in the age group ranging from 32 to 43 years. This could be because this population represents a more responsible set of individuals who may be more organized and motivated in improving their health and lifestyle. This result is in contrary to the findings from a study performed to analyze adherence among older HIV infected patients and in a prospective cohort study in Cameroon. They reported that older patients (> 50 years) showed higher tolerance to ART and young patients (< 36 years) showed poorer adherence [22, 26]. Furthermore, females were better adherent to ART. This could be due to the fact that women more often attend health care fascilities, compared to men. Consequently, a high proportion of women were represented in our study among those adherent to ART.

Limitations of the study include the loss to follow-up of patients, as that might have influenced the efficacy of our findings. In addition, we evaluated adherence based on pharmacy records, on the number of patients who continued treatment, and did not investigate the correlation or association between number of doses, adherence and viral outcome. Our findings add evidence to the need of improving strategies to evaluate adherence in HIV patients on ART. Large cohort studies are needed to validate these findings and evaluate the different methods used for assessing adherence to ART. It is important to investigate the various factors influencing adherence. The results from these studies can be incorporated to enhance the management of HIV infected individuals on ART.

## Conclusion

Adherence and patient counselling play an important role in maintaining successful ART outcomes. Ineffective ART induces virological failure. There was a significant decrease in the viral load suppression in both regimens. The EFV regimen was more effective in suppressing viral load and is less likely to induce a virological failure response, compared to the NVP regimen.

## Abbreviations

3TC: Lamivudine
ART: Antiretroviral therapy
AZT: Zidovudine
CSCCD: Center for the Study and Control of Communicable Diseases
EDTA: Ethylenediaminetetraacetic acid
EFV: Efavirenz
MEMS: Medication event monitoring systems
NNRTIs: Non-nucleoside reverse transcriptase inhibitors
NRTIs: Nucleoside reverse transcriptase inhibitors
NVP: Nevirapine
RAMs: Resistance associated mutations
TDF: Tenofovir
